# Women’s techniques for making vaginal penetration more pleasurable: Results from a nationally representative study of adult women in the United States

**DOI:** 10.1371/journal.pone.0249242

**Published:** 2021-04-14

**Authors:** Devon J. Hensel, Christiana D. von Hippel, Charles C. Lapage, Robert H. Perkins

**Affiliations:** 1 OMGYES Research Group, For Goodness Sake LLC, Berkeley, CA, United States of America; 2 Department of Pediatrics, Division of Adolescent Medicine, Indiana University School of Medicine, Indianapolis, IN, United States of America; 3 Department of Sociology, Indiana University Purdue University-Indianapolis, Indianapolis, IN, United States of America; University of Westminster, UNITED KINGDOM

## Abstract

The study purpose was to assess, in a U.S. probability sample of women, the specific ways women have discovered to make vaginal penetration more pleasurable. Through qualitative pilot research with women that informed the development of the survey instrument used in this study, we identified four previously unnamed, but distinct, techniques women use to make vaginal penetration more pleasurable: Angling, Rocking, Shallowing and Pairing. This study defines each technique and describes its prevalence among U.S. adult women. Weighted frequencies were drawn from the Second OMGYES Pleasure Report—a cross-sectional, online, national probability survey of 3017 American women’s (age 18–93) sexual experiences and discoveries. Participants were recruited via the Ipsos KnowledgePanel®. Data suggest that 87.5% of women make vaginal penetration more pleasurable using ‘Angling’: rotating, raising, or lowering the pelvis/hips during penetration to adjust where inside the vagina the toy or penis rubs and what it feels like. Approximately 76% of women make vaginal penetration more pleasurable using ‘Rocking’: the base of a penis or sex toy rubbing against the clitoris constantly during penetration, by staying all the way inside the vagina rather than thrusting in and out. About 84% of women make vaginal penetration more pleasurable using ‘Shallowing’: penetrative touch just inside of the entrance of the vagina—not on the outside, but also not deep inside—with a fingertip, sex toy, penis tip, tongue, or lips. Finally, 69.7% of women orgasm more often or make vaginal penetration more pleasurable using ‘Pairing’: when a woman herself (Solo Pairing) or her partner (Partner Pairing) reaches down to stimulate her clitoris with a finger or sex toy at the same time as her vagina is being penetrated. These data provide techniques that are at women’s disposal to make penetration more pleasurable—which can enable women to better identify their own preferences, communicate about them and advocate for their sexual pleasure.

## Introduction

This paper engages nationally representative data to understand U.S. adult women’s preferences for vaginal stimulation and penetration techniques as a means of increasing sexual pleasure. Holistic approaches to sexual health increasingly emphasize the positive contributions that sexual pleasure–particularly for women–provides to physical, social and emotional well-being [1, 2] across the lifespan [3, 4]. For example, research has shown that sexual pleasure contributes to women’s reports of greater happiness, and lower levels of depression, stress and anxiety [5–7]. Literature has also linked sexual pleasure within partnerships to greater relationship satisfaction, intimacy, and commitment for women [7–10].

Viewing sexual pleasure as a critical scaffold to women’s well-being is important because it reframes their enjoyment of sex as a fundamental human right, rather than as a medical or psychological problem to be “solved” [11, 12]. Several international sexual and reproductive health organizations, including The World Association for Sexual Health (WAS) and the International Planned Parenthood Federation (IPPF), explicitly include “pleasurable” and “satisfying” sexual experiences within their declarations on sexual rights [13]. A pleasure-as-rights orientation is a *person-centered* way of approaching sexual pleasure. Person-centered perspectives seek to understand the subjectivity of each woman’s lived experiences of pleasure, including the development of skills—such as communication, confidence, and the ability to negotiate with partners—that increase her agency to access enjoyable sex [11, 14, 15]. Sexual pleasure research can support person-centered perspectives by assessing granularity of what makes sex enjoyable for *each* women, rather than making the assumption that the same handful of approaches work equally well for *all* women [16–18].

However, despite the increased interest in supporting a more person-centered approach in women’s sexual pleasure research, much of the extant scientific literature lacks the detailed information necessary to foster a more comprehensive understanding of how pleasure experiences are organized. Most of the existing research broadly focuses on the *body part or object* that stimulates or penetrates the vagina (e.g. a penis, a hand/finger, or a sex toy, etc.) [19], rather than documenting the specific vaginal stimulation and penetration *techniques* women themselves report using for pleasure [20], or any technique innovations, or variations, that women use to make them more pleasurable or easier for them to use on their own or with a partner [21] For example, studies from the United States, Canada, Australia and Germany have shown that vaginal use of vibrators, dildos and other sex toys among heterosexual, gay/lesbian and bisexually identified adult women are associated with greater sexual pleasure and satisfaction during solo or partnered sex [22–25]. Other work engaging adult samples from the United States, China, Australia and Sweden has examined the extent to which a wider sexual repertoire, including both penile-vaginal sex and noncoital sexual behaviors, such as giving/receiving oral sex or manual stimulation, is associated with greater likelihood of women’s orgasm, sexual satisfaction or sexual enjoyment [26–32]. A smaller number of studies have also investigated bondage, domination, submission/sadism, and masochism (BDSM) or “kink” vaginal penetration behaviors (e.g. fisting or stretching) as a source of sexual arousal or eroticism [25, 33, 34]. Neither the sex toy- nor the sexual behavior-focused bodies of work elicited more granular information from participants about specific ways in which techniques were used to increase pleasure.

Other research investigates the extent to which body positioning (e.g. the “coital alignment technique” as well as more colloquially known terms like “face-to-face” or “rear entry”) matters for women’s sexual pleasure [35–37]. This body of research, however, is limited to penile-vaginal penetrative acts between a man and a woman and does not provide more detailed information about how variations in body movement (e.g., staying inside vs. thrusting, rotating vs. remaining still) may be associated with sexual pleasure. An additional line of research has documented the extent to which manual clitoral stimulation is done simultaneously with penile-vaginal penetration to augment pleasure and orgasm [38–40], but this research has not fully examined whether this clitoral stimulation is woman-controlled, partner-controlled or a combination of the both, nor has it examined whether the clitoral stimulation is done with a finger or sex toy.

Absent detailed and robust person-centered empirical data on women’s sexual pleasure approaches in the scientific literature, women and/or their partners may be left to rely on non-scientific sources, such as popular print or electronic media, to inform their expectations about which stimulation and penetration techniques they can and should use to enjoy sex. Studies on these non-scientific sources suggest that they often focus on a handful of the “most effective” or “right” techniques [41], often in slang (e.g. “finger banging”) [42, 43] or ambiguous terms (e.g. “foreplay” or “outercourse”) [44, 45] or overly clinical terms (“manual-genital stimulation” or “G-spot stimulation”) [19, 46, 47] Common terms rarely acknowledge a wider repertoire of vaginal stimulation and penetration techniques that women can and do use for pleasure as part of their solo and partnered sex lives [48].

Collectively, neither popular media nor current scientific literature fully represent the range of vaginal stimulation and penetration techniques women and partners use or the details of how they tweak and personalize the techniques they use in order to enhance their pleasure. A lack of both educational and informational alternatives to these existing sources matters because optimizing sexual health means that individuals need to be empowered to seek out the pleasure promoting techniques that meet *their* needs, rather than falling back on what literature/media tells them they “should” like or what they expect is “most likely” to be enjoyable [12].

Nationally representative studies–with a focus on detailed sexual touch and stimulation techniques–are a powerful method to fill the identified gaps. From a *measurement* perspective, inclusion of a range of penetration variants currently unexplored in peer-reviewed research provides a means by which to validate the range of pleasure approaches that women use in daily life. Researchers can provide data that identify multiple techniques, give those techniques names, and develop descriptions of how the application of a given technique differs from that of another. Such specificity has the potential to directly address the ambiguity and efficiency challenges of existing language used to describe vaginal stimulation and penetration. For example, as described earlier, ambiguous, slang-based, or overly clinical terms [19, 41–47, 49] are often used in scientific and popular media to describe stimulating or penetrating the vagina. Inaccessible vocabulary can present substantial barriers to a woman’s ability to accurately describe to a partner *what* techniques she would like to use, and *how* she would like them performed [50]. This ambiguity could likewise impede a woman’s individual learning of how to amend existing sexual techniques she likes, or to learn new techniques. In addition, accurate language is important because the words people use to describe an activity help shape the meaning they ascribe to it [32]. A woman having access to a wider stimulation and penetration vocabulary may increase her satisfaction with her sexual communication with partners [51, 52], which in turn may have positive implications for both relationship satisfaction and sexual satisfaction [53–55].

From a *sampling* perspective, sexual pleasure literature has typically been conducted with convenience or community samples, samples drawn from clinical populations, or from college campuses [26, 56–59]. Several nationally representative studies document generally link a range of sexual behaviors to sexual pleasure [27–31], but they do not use detailed stimulation and penetration technique measures. Engaging large, nationally representative studies that include these detailed items allows researchers to evaluate both the prevalence of and patterns within specific pleasure approaches in ways that reflect the experiences of all women in a population. This presentation is helpful for individuals to be able to see their own preference(s) normalized within a range of other techniques, as well as to see the extent to which their experiences are shared by their peer group.

These measurement and sampling strengths of nationally representative studies are exemplified in *the first OMGYES Pleasure Report—*a nationally representative study that members of our team conducted to examine, in detail, American adult women’s experiences with and preferences for *external* genital touch and stimulation [49]. Rather than asking participants whether or not they liked to be touched externally, the study demonstrated that women use different *ways* of touching–different locations, pressure, shapes and patterns–as a means of increasing their sexual enjoyment. The authors noted that assessing the range and complexity of external touch dimensionality in peer-reviewed literature normalizes the specific, but different axes on which “typical” women engage external touch, and at an individual level, provides specific and actionable ideas that women and partners can choose to integrate into their own repertoire. Such data illustrate the need for ongoing studies to provide data to document sexual pleasure pathways.

Accordingly, the purpose of the current study was to use nationally representative probability data–*the second OMGYES Pleasure Report*–to investigate U.S. women’s preferences for *internal* touch, specifically vaginal stimulation and penetration.

## Materials and methods

### Survey development

The design of survey items for the second OMGYES Pleasure Report began with a large scale, exploratory qualitative study (2014–2015; IRBs # 2003603806 and 2004356627) to broadly generate information about women’s discoveries and experiences with genital stimulation and sexual pleasure. We conducted a series of initial, broad, online surveys of 4270 adult (18+) women from around the world, recruited through social media advertisements. Potential participants clicked on a link to the survey in the advertisement and responded in text boxes to open-ended questions such as “What discovery have you made that really made vaginal penetration more pleasurable for you?” A subset of approximately 1000 women participated in follow-up interviews conducted via video chat. These secondary, semi-structured interviews were focused on eliciting more detail about the specific strategies, insights, and techniques respondents had found pivotal to increasing their sexual pleasure. Interviews ranging between 15 and 60 minutes in length, were conducted and recorded by OMGyes researchers. Data from these interviews were analyzed by OMGyes research staff and consultants using an inductive constructivist thematic analysis framework to identify where and/or how similarities emerged in the ways in which women enjoy specific aspects of sexual touch and vaginal stimulation [60]. This approach of identifying, analyzing, and reporting patterns/themes is particularly suitable for nuanced exploratory work in understudied areas [61] Numerous techniques that women use to enhance their pleasure during penetration emerged—most under described in the current literature; the present analysis focuses on four of these techniques. We searched existing scientific and popular sexual pleasure literature for established terms that described those four technique forms, and we were unable to find such existing language. Therefore, we gave each form a descriptive name—**Angling**, **Rocking**, **Shallowing**, and **Pairing**. Definitions and sexually explicit line drawing illustrations of these four techniques are provided in S1 Table. Images contained in this table are visually graphic.

In the next stage of development, and the focus of the current study, was the development of a quantitative survey to investigate the population level prevalence of women who report using the Angling, Rocking, Shallowing and Pairing and their sub-forms. The description of all items on our survey is presented in the next section. All items are original to this study—as informed by our exploratory qualitative work—and have not been yet examined in the peer-reviewed literature.

### Data collection

All study procedures were approved by the institutional review board at Indiana University School of Medicine (IRB # 1801846511). Data were drawn from the second OMGYES Pleasure Report—a cross-sectional, online, nationally representative survey of sexual behaviors, sexual attitudes, relationships, sexual satisfaction, and experiences with genital touching among women aged 18 and over in the United States. The study was conducted in July 2018 by Ipsos Research using their KnowledgePanel® (Menlo Park, California) to recruit a probability-based web panel designed to be representative–including an oversample of lesbian and bisexual women–of all noninstitutionalized U.S. women. Ipsos creates research panels using an address-based sampling (ABS) frame from using the U.S. Postal Service’s Delivery Sequence File–a database with full coverage of all delivery points in the US. ABS not only improves population coverage, but also provides a more effective means for recruiting hard-to-reach individuals, such as young adults, those without landline telephones, and minorities. Panel member households without internet connection are provided with a web-enabled device and free Internet service to maximize the breadth of participation.

The 90-item online survey took a median of 29 minutes to complete, was available in English and Spanish languages, and was open for participation from July 12-July 31, 2018. Questions assessed participation in demographics, sexual behavior background, as well as lifetime participation in different types of Angling, Rocking, Shallowing, and Pairing techniques. Participants could also access clickable pop-up illustrations provided for some techniques within the survey. Panel households (N = 6123) randomly selected to participate were sent an email that informed them of the survey’s availability and provided them a link to the survey. KnowledgePanel® members typically only receive one survey per week, with an average of two to three per month. Reminder emails were sent to survey non-responders (on Days 3, 9, 12, 15 and 18) during the study period. Of the original households recruited (N = 6123), 3398 (55.5%) opened the study link, where they were able to view detailed information about the study. If they wanted to participate, they could open an informed consent statement to read. Those who wanted to continue clicked “I agree” to indicate electronically recorded consent with Ipsos. Of those who opened the study link, 88.8% (3017/3398) completed the survey (49.7% [3017/6123] of the initial sampling frame) and represent the analytical sample in this study. This completion rate is similar to other Ipsos-conducted nationally representative studies of sexuality and sexual behavior (44% - 51%) [32, 50, 51, 62]. Ipsos operates a modest incentive program that includes raffles and sweepstakes with both cash rewards and other prizes for completing the survey.

Ipsos provided post-stratification, study-specific weights to adjust for any over- or under-sampling as well as non-response. Distributional data pertinent to the study population were obtained either from the U.S. Census Bureau’s American Community Survey (ACS), or from the weighted KnowledgePanel® profile data. Using this information, final weights were calculated using an iterative proportional fitting procedure, and if necessary, were trimmed at the extreme upper and lower tails of the weight distribution. The resulting weights were then scaled to aggregate to the total sample size of all eligible respondents. Weighted participant characteristics are described in Table 1. All data used in this study are available through the ICPSR open data sharing consortium [63].

**Table 1:** Demographics of national probability sample of 3017 U.S. women (weighted).

Demographics	n	Percentage
**Sexual Orientation**		
Gay or Lesbian	68	2.2%
Bisexual	176	5.8%
Something else	21	0.7%
Straight/heterosexual	2752	91.2%
**Gender Identity**		
Cisgender	2973	98.5%
Transgender	10	0.3%
Something else	8	0.3%
Missing	26	0.9%
**Relationship Status**		
Single and not dating	652	21.6%
Single and dating or hanging out with someone	166	5.5%
In a relationship but not living together	215	7.1%
Living together but not married	309	10.2%
Married	1653	54.8%
In more than one relationship	16	0.5%
Missing	6	0.2%
**Age**		
18–29	590	19.6%
30–44	792	26.2%
45–59	789	26.2%
60+	846	28.0%
**Race and Ethnicity**		
White, non-Hispanic	1925	63.8%
Black, non-Hispanic	376	12.4%
Other Race, non-Hispanic	207	6.8%
Hispanic	468	15.5%
>2 Races, non-Hispanic	42	1.4%
**Education**		
Less than High School	301	10.0%
High School	815	27.0%
Some College	911	30.2%
Bachelor’s Degree or Higher	990	32.8%
**Household Income**		
Under $25,000	504	16.6%
$25,000–$49,999	624	20.7%
$50,000–$74,999	523	17.4%
$75,000–$99,999	412	13.6%
$100,000–$149,999	479	15.9%
$150,00 and over	475	15.7%
**Geographic Region of Residence**		
Northeast	531	17.6%
Midwest	634	21.0%
South	1150	38.1%
West	701	23.2%

### Measures

#### Angling measures

Questions about Angling were prefaced in the survey with a statement that said: “Many women have discovered that, during vaginal intercourse with a toy or penis, the angle or position of their hips makes a big difference in where the toy or penis rubs and what it feels like.” Participants then indicated through yes/no responses to 6 items whether or not they had ever used sub-forms of Angling “to make sex better / more pleasurable during penetration.” An affirmative response to any of the 6 items indicated a woman had used any form of Angling to enhance her pleasure during vaginal penetration. An affirmative response to one or more of three specific items (“rotating hips downward while on back;” “rotating hips upward while lying on stomach;” “putting a pillow under lower back so pelvis is lower”) (all: no/yes) indicated use of the sub-form of Angling referred to as ‘Angling hips low;’ these three items were grouped into this sub-form category because the direction of rotation of the pelvis (colloquially ‘hips’) is mechanically equivalent whether performed while lying on one’s stomach or back (with or without a pillow to support rotation). Similarly, an affirmative response to one or more of the three remaining specific items (“rotating hips upward while lying on back;” “rotating hips downward while lying on stomach;” “putting a pillow under butt so pelvis is higher”) (all: no/yes) indicated use of the sub-form of Angling referred to as ‘Angling hips high;’ again, these three items were grouped into this sub-form category because the direction of rotation of the pelvis/hips is mechanically equivalent whether performed while lying on one’s stomach or back (with or without a pillow to support rotation).

#### Rocking measures

Questions about Rocking asked women about the extent to which they had ever found pleasurable (all: four-point Likert scale: not pleasurable to very pleasurable; or “don’t know or never tried”) two different methods of penetration in which the base of a partner’s penis or a sex toy rubbed against the clitoris constantly during penetration, by staying all the way inside the vagina rather than thrusting in and out. The sub-form of Rocking done with a penis (‘Rocking with a penis’) was assessed with the following item: “a partner’s penis “staying inside” with their lower body staying in contact and pressing against your clitoris.” The sub-form of Rocking done with a sex toy (‘Rocking with a sex toy’) was assessed with the following item: “a toy “staying inside” your vagina without in-and-out thrusting.” All items were dichotomized (not at all/a little pleasurable vs. somewhat/very pleasurable) for analysis and women who answered “don’t know or never tried” were excluded. A response of somewhat/very pleasurable to at least one Rocking item indicated a woman had used any form of Rocking to enhance her pleasure during vaginal penetration.

#### Shallowing measures

Questions about Shallowing asked women about the extent to which they had ever found pleasurable (all: four-point Likert scale: not pleasurable to very pleasurable; or “don’t know or never tried”) different methods of being touched “just inside, at the entrance of your vagina (just shallow penetration—not on the outside, but also not deep inside).” The four items relating to specific Shallowing sub-forms were: “with a fingertip,” “with a sex toy,” “with the tongue or lips,” and “with the tip of the penis.” All items were dichotomized (not at all/a little pleasurable vs. somewhat/very pleasurable) for analysis and women who answered “don’t know or never tried” were excluded. A response of somewhat/very pleasurable to at least one Shallowing item indicated a woman had used any form of Shallowing to enhance her pleasure during vaginal penetration.

Women who had used any form of Shallowing to enhance their pleasure from penetration were also asked whether Shallowing changed their sexual pleasure in different scenarios. Participants were shown the statement: “Some women say that having the tip of a penis, sex toy, or fingers just inside the entrance to the vagina (with shallow penetration), even briefly, changes how pleasurable the sex or sexual touching that follows feels.” Women then indicated whether any of the following pleasure experiences had occurred (all: no/yes) for them during sexual stimulation after Shallowing: “the penetration that comes next is more likely to be pleasurable;” “the orgasm that comes next is more likely to be stronger or more intense;” “the orgasm that comes next is more likely to happen at all;” “it depends—there is no consistent pattern.”

#### Pairing measures

Questions about Pairing asked women to indicate through yes/no responses to 4 items whether or not they had ever used sub-forms of Pairing (i.e. specific forms of clitoral touch in combination with vaginal penetration) to “orgasm more often or have more pleasurable sex” than from using penetration on its own. Sub-forms of Pairing where the clitoral touch was done by the woman herself (‘Solo Pairing’) were assessed with the following 2 items (all: no/yes) “penetration while stimulating my own clitoris at the same time with a finger;” “penetration while stimulating my own clitoris at the same time with a toy or vibrator.” Sub-forms of Pairing where the clitoral touch was done by the woman’s partner (‘Partner Pairing’) were assessed with the following items (all: no/yes): “penetration with my partner stimulating my clitoris at the same time with a finger;” “penetration with my partner stimulating my clitoris at the same time with a toy or vibrator.” An affirmative response to any of the 4 items used to assess sub-forms of Pairing indicated a woman had used any form of Pairing to enhance her pleasure during vaginal penetration.

### Statistical procedure

Weighted frequencies were calculated to assess the prevalence of women who have used Angling, Rocking, Shallowing, Pairing, and their sub-forms to make vaginal stimulation and penetration more pleasurable. We excluded from analysis of each item any participant whose response to that item was missing. IBM SPSS Statistics software was used for all analyses.

## Results and discussion

### Respondent characteristics

Weighted respondent demographic characteristics—including age, gender, race/ethnicity, education, household income, and geographic region of residence in the US, sexual orientation and relationship status—are presented in Table 1. Women ranged in age from 18 to 93 with a median age of 48 years. The majority of women self-described their sexual orientation as heterosexual (91.2%). Most women were in a married, committed, or dating relationship, with only 21.6% describing their relationship status as single and not dating at the time of the survey.

### Prevalence of and pleasure during Angling

As shown in Table 2, 87.5% of women had ever used any form of Angling (i.e., adjusting pelvic—or colloquially, ‘hip’—height or rotation while lying on one’s back or stomach) to increase their sexual pleasure during vaginal penetration. About two-thirds (67.6%) of women had used the sub-form of ‘Angling hips low’ to enhance their pleasure from penetration. A slightly greater proportion of women (83.5%) of women had used the sub-form of ‘Angling hips high’ to enhance their pleasure from penetration.

**Table 2:** Prevalence of use of Angling techniques by women to make vaginal penetration more pleasurable (N = 2898, weighted).

	Percentage of women who have used this technique to make penetration more pleasurable (n)
**Any form of Angling**	87.5% (2535)
**Sub-forms of Angling:**	
**Angling hips low**	67.6% (1958)
**Angling hips high**	83.5% (2420)

### Prevalence of and pleasure during Rocking

As shown in Table 3, 76.4% of women had ever used any form of Rocking (i.e. the base of a penis or sex toy rubbing against the clitoris constantly during penetration, by staying all the way inside the vagina rather than thrusting in and out) to enhance their pleasure during vaginal penetration. Nearly three-quarters of women (70.2%) had ever engaged in Rocking with a penis to make penetration more pleasurable for themselves. In contrast, one-third of women had ever used Rocking with a sex toy (35.8%), to make penetration more pleasurable.

**Table 3:** Prevalence of use of Rocking techniques by women to make vaginal penetration more pleasurable (N = 2844, weighted).

	Percentage of women who have used this technique to make penetration more pleasurable (n)
**Any form of Rocking**	76.4% (2172)
**Sub-forms of Rocking:**	
**Rocking with a penis**	70.2% (1997)
**Rocking with a sex toy**	35.8% (1017)

### Prevalence of and pleasure during Shallowing

As shown in Table 4, approximately 4 out of 5 women (83.8%) had ever used any form of Shallowing (e.g. being touched just at the entrance to the vagina) to enhance their pleasure during penetration. A majority of women had ever done Shallowing with a penis tip (67.1%) or with a tongue or lips (69.7%) to make penetration more pleasurable. A slightly smaller proportion of women had used Shallowing with a fingertip (60.4%) to enhance their pleasure from penetration. Shallowing was less commonly done with a sex toy (37.8%).

**Table 4:** Prevalence of use of Shallowing techniques by women to make vaginal penetration more pleasurable (N = 2939, weighted).

	Percentage of women who have used this technique to make penetration more pleasurable (n)
**Any form of Shallowing**	83.8% (2463)
**Sub-forms of Shallowing:**	
**Shallowing with a tongue or lips**	69.7% (2047)
**Shallowing with a penis tip**	67.1% (1971)
**Shallowing with a fingertip**	60.4% (1776)
**Shallowing with a sex toy**	37.8% (1111)

Table 5 illustrates the ways in which women reported that using a Shallowing technique enhanced their sexual pleasure during penetration that followed Shallowing. About 40% of women noted more pleasure, while a quarter reported stronger orgasms. Only 10.1% thought that their likelihood of orgasm was increased. One-third of women (37.4%) suggested that their pleasure was impacted, but in a way that did not follow a specific pattern.

**Table 5:** Prevalence of specific types of pleasure enhancement experienced by women during vaginal penetration following Shallowing (N = 2723, weighted).

	Percentage of women who experienced this type of pleasure enhancement during penetration following Shallowing (n)
**More pleasure**	40.2% (1096)
**Stronger orgasm**	25.3% (690)
**Increased likelihood of orgasm**	10.1% (276)
**Pleasure is affected, but not in a specific manner**	37.4% (1017)

### Prevalence of and pleasure during Pairing

As shown in Table 6, 69.7% of women have ever used a Solo Pairing or Partner Pairing technique (e.g. woman stimulating her clitoris with a finger or a partner doing so while the partner simultaneously penetrates her vagina) to make penetration more pleasurable. Forty percent of women had used Solo Pairing to make penetration more pleasurable; about one-third of women had ever done so with a finger, and 20% had ever done so with a sex toy. About half of women had engaged in Partner Pairing to make penetration more pleasurable (49%); Partner Pairing was more commonly done with a finger (43.1%) than with a sex toy (17.5%).

**Table 6:** Prevalence of use of Pairing techniques by women to make vaginal penetration more pleasurable (N = 2877, weighted).

	Percentage of women who have used this technique to make penetration more pleasurable (n)
**Any form of Pairing**	69.7% (2006)
**Sub-forms of Solo Pairing**	40.0% (1152)
**Solo Pairing with a finger**	30.9% (890)
**Solo Pairing with a sex toy**	20.1% (578)
**Sub-forms of Partner Pairing**	49.0% (1410)
**Partner Pairing with a finger**	43.1% (1239)
**Partner Pairing with a sex toy**	17.5% (504)

## Discussion

The purpose of this paper was to use U.S. nationally representative probability data to describe specific techniques women use to make vaginal penetration more pleasurable. Because sexual pleasure is a critical element in women’s health and well-being across the life span [1–4], there have been several calls to better understand the subjectivity with which women access and experience enjoyable sex [64]. Extant literature, however, has generally failed to capture the specific approaches that women actually use in everyday life as vehicles to enjoy sex [20, 21]. Using data from the second OMGYES Pleasure Report, this study directly addresses this gap by providing the first detailed, population level description of the of the distinct vaginal stimulation and penetration techniques women engage to enhance their sexual pleasure during both solo and partnered sex. Specifically, through inductive qualitative research, we identified four previously unnamed, but distinct, forms of vaginal stimulation and penetration—Angling, Rocking, Shallowing and Pairing—and demonstrated that each, as well as the sub-forms for engaging them, are prevalent among U.S. adult women as a means of making sex more pleasurable.

Capturing the granularity of women’s sexual enjoyment is consistent with a person-centered perspective on sexual pleasure. Such a perspective places emphasis on understanding the ways in which women can scaffold specific skills to increase their access to pleasure [11, 14, 15]. For example, as suggested earlier in this paper, most of what is known—either scientifically or through popular culture—about “how” to access sexual pleasure is focused on the object or body part penetrating the vagina [22–25], a specific body position or sexual behavior [26–32, 35–37], or vague/imprecise terminology [19, 41–47]. The collective gaps left in this knowledge may prevent women and/or their partners from knowing the range of different approaches that exist or how to use them. An important contribution of this work is our provision of actual names/terms for vaginal stimulation and penetration forms as well as our specific yet lay-interpretable definitions and illustrations to describe them. Increasing the availability and accessibility of sexual pleasure language—straightforward and comfortable words and descriptions that women can use [49] - can help validate for women *what* they like from vaginal stimulation and penetration and *how* they would like those techniques performed [12, 65]. This accessible sexual pleasure language could also help provide women and their partners novel ways of considering and communicating about having new stimulation/penetration experiences together [48]. Such increased communication efficacy may have additional downstream, positive implications for both relationship satisfaction and sexual satisfaction [53–55]. The building of these skills—communication, confidence and negotiation—is resonant with a person-centered framework [11, 14, 15].

An additional limitation contributed by a lack of extant, detailed research on women’s sexual pleasure techniques means that women may use a narrow, socially constructed model as a means to judge their own subjective experiences [16–18]. Our use of nationally representative data permits us to provide a population level analysis of each vaginal stimulation and penetration technique we assessed. Data such as ours provide a means through which women can more fully contextualize the range of their own lived experiences with vaginal penetration. Our assessment of 14 different sub-forms within our four larger Angling, Rocking, Shallowing and Pairing forms—*as informed by the voices of women themselves*—validates both that adult women choose a range of different approaches to vaginal stimulation and penetration to increase their sexual pleasure, and that women actively choose different movements and angles to personalize these approaches. Moreover, we examined the prevalence of each stimulation and penetration technique and their sub-forms and we demonstrated that most women—typically greater than 70%—have found a given technique enhances their sexual pleasure. Our provision of bottom-up generated, population level information supports a larger call for research to more holistically capture what makes sex enjoyable for women [66]. Information such as this allows women to situate and “normalize” their own preferences within the diverse repertoire noted by other women, as well to see that there may be even more techniques for enhancing pleasure during vaginal stimulation and penetration for them to explore.

### Limitations and strengths

There are several limitations associated with these data. Our survey was limited to women; we did not survey men to ask about their experiences with vaginal stimulation and penetration techniques and any perceived impacts on their own or their partner’s pleasure. Moreover, we did not include information on how relationship factors may influence women’s participation in and/or their enjoyment of specific techniques. Future work may seek to recruit both dyad members in a relationship as a means of contextualizing a partner’s role in the technique selection and experience process. Furthermore, some survey items assessed technique participation in general, whereas others assessed technique participation in association with sexual pleasure, which could challenge disentangling a participant’s reported use of technique from their motivation for choosing that technique, as well as disentangling their expectation of pleasure from their actual experience of pleasure. A future solution to increase measurement precision could be to assess overall frequency and/or lifetime reports in specific techniques, and to engage contingent questions regarding motivation or expected pleasure, as well as actual pleasure outcomes for those who affirmed participation.

These limitations are balanced with several methodological and substantive strengths of this study. From a methodological perspective, our use of a nationally representative probability sample permits generalization of findings to the broader population of adult women in the United States. Other sampling approaches common in sexual and reproductive health research, including convenience, clinical or community-based recruitment, do not allow this level of comparison. In addition, our use of Ipsos’ KnowledgePanel® affords several data collection advantages, including access to already experienced survey participants, secure survey storage and sending of participation reminders to potential respondents. Ipsos also controls the number of surveys sent to each member, minimizing the unit- and item-level missingness on any given survey. Another methodological strength is online data collection, which facilitates survey completion in a setting of the participant’s choosing, thereby increasing data confidentiality and participant comfort with answering questions about potentially sensitive topics, like sexual behavior and sexual pleasure.

## Conclusion

Data from this U.S. nationally representative survey provide descriptions of and prevalence estimates for four techniques women have discovered to make vaginal penetration more pleasurable: Angling, Rocking, Shallowing, and Pairing. Our findings contribute to the growth of a person-centered approach to sexual pleasure, previously underexplored in published literature, through detailed documentation of specific, newly identified forms of vaginal stimulation and penetration, as well as evaluation of technique prevalence. Knowledge of these techniques can enable women to better identify their own preferences, communicate about them and advocate for their sexual pleasure.

## Supporting information

S1 Table Definitions for and sexually explicit line drawing illustrations of Angling, Rocking, Shallowing and Pairing. (DOCX)

S1 Appendix (DOCX)

## References

[1] Diamond LM, Huebner DM. Is Good Sex Good for You? Rethinking Sexuality and Health. Social and Personality Psychology Compass. 2012;6(1):54–69.

[2] Hensel DJ, Nance J, Fortenberry JD. The association between sexual health and physical, mental, and social health in adolescent women. Journal of Adolescent Health. 2016. 10.1016/j.jadohealth.2016.06.003 PMC8596160 27491340

[3] Hensel DJ, Fortenberry JD. Lifespan Sexuality through a Sexual Health Perspective. In: Tolman DL, Diamond LM, editors. APA Handbook on Sexuality and Psychology. Washington, D.C.: APA Press; 2013.

[4] World Association for Sexual Health. Achieve recognition of sexual pleasure as a component of well-being. In: World Association for Sexual Health, editor. Millenium declaration. Oaxaca, Mexico 2008.

[5] Davison SL, Bell RJ, LaChina M, Holden SL, Davis SR. The Relationship between Self-Reported Sexual Satisfaction and General Well-Being in Women. The Journal of Sexual Medicine. 2009;6(10):2690–7. 10.1111/j.1743-6109.2009.01406.x 19817981

[6] O’Leary KD, Acevedo BP, Aron A, Huddy L, Mashek D. Is long-term love more than a rare phenomenon? If so, what are its correlates? Social Psychological and Personality Science. 2012;3(2):241–9.

[7] Sánchez-Fuentes MdM, Santos-Iglesias P, Sierra JC. A systematic review of sexual satisfaction. International Journal of Clinical and Health Psychology. 2014;14(1):67–75.

[8] McNulty JK, Wenner CA, Fisher TD. Longitudinal associations among relationship satisfaction, sexual satisfaction, and frequency of sex in early marriage. Archives of Sexual Behavior. 2016;45(1):85–97. 10.1007/s10508-014-0444-6 25518817 PMC4472635

[9] Sprecher S, Cate RM, Harvey J, Wenzel A. Sexual satisfaction and sexual expression as predictors of relationship satisfaction and stability. The handbook of sexuality in close relationships. 2004:235–56.

[10] Yoo H, Bartle-Haring S, Day RD, Gangamma R. Couple communication, emotional and sexual intimacy, and relationship satisfaction. Journal of Sex & Marital Therapy. 2014;40(4):275–93. 24111536 10.1080/0092623X.2012.751072

[11] Gruskin S, Yadav V, Castellanos-Usigli A, Khizanishvili G, Kismödi E. Sexual health, sexual rights and sexual pleasure: meaningfully engaging the perfect triangle. Sexual and Reproductive Health Matters. 2019;27(1):29–40. 10.1080/26410397.2019.1593787 31533569 PMC7887957

[12] Wampold CH. The components of great sex: Sexuality education for people who desire to scale the heights of optimal sexuality. American Journal of Sexuality Education. 2014;9(2):219–28.

[13] Giami A. Sexuality, health and human rights: The invention of sexual rights. Sexologies. 2015;24(3):e45–e53.

[14] Hirst J. ‘It’s got to be about enjoying yourself’: young people, sexual pleasure, and sex and relationships education. Sex Education. 2013;13(4):423–36.

[15] Hull TH. Sexual Pleasure and Wellbeing. International Journal of Sexual Health. 2008;20(1/2):133–45.

[16] Arcos-Romero AI, Sierra JC. Factors associated with subjective orgasm experience in heterosexual relationships. Journal of Sex & Marital Therapy. 2020;46(4):314–29. 10.1080/0092623X.2019.1711273 31914865

[17] Cherkasskaya E, Rosario M. The relational and bodily experiences theory of sexual desire in women. Archives of Sexual Behavior. 2019;48(6):1659–81. 10.1007/s10508-018-1212-9 29926262

[18] Pascoal PM, Narciso IdSB, Pereira NM. What is sexual satisfaction? Thematic analysis of lay people’s definitions. Journal of sex research. 2014;51(1):22–30. 10.1080/00224499.2013.815149 24070214

[19] Schick V, Herbenick D, Rosenberger JG, Reece M. Variations in the Sexual Repertoires of Bisexually-Identified Women from the United States and the United Kingdom. Journal of Bisexuality. 2012;12(2):198–213.

[20] Ford JV, Corona Vargas E, Finotelli I Jr, Fortenberry JD, Kismödi E, Philpott A, et al. Why Pleasure Matters: Its Global Relevance for Sexual Health, Sexual Rights and Wellbeing. International Journal of Sexual Health. 2019:1–14.

[21] Starrs AM, Ezeh AC, Barker G, Basu A, Bertrand JT, Blum R, et al. Accelerate progress—sexual and reproductive health and rights for all: report of the Guttmacher–Lancet Commission. The Lancet. 2018. 10.1016/S0140-6736(18)30293-9 29753597

[22] Herbenick D, Reece M, Sanders S, Dodge B, Ghassemi A, Fortenberry JD. Prevalence and characteristics of vibrator use by women in the United States: Results from a nationally representative study. The journal of sexual medicine. 2009;6(7):1857–66. 10.1111/j.1743-6109.2009.01318.x 19453881

[23] Döring N, Poeschl S. Experiences with diverse sex toys among German heterosexual adults: Findings from a national online survey. The Journal of Sex Research. 2020;57(7):885–96. 10.1080/00224499.2019.1578329 30806076

[24] Wood J, Crann S, Cunningham S, Money D, O’Doherty K. A cross-sectional survey of sex toy use, characteristics of sex toy use hygiene behaviours, and vulvovaginal health outcomes in Canada. The Canadian Journal of Human Sexuality. 2017;26(3):196–204.

[25] Richters J, Grulich AE, de Visser RO, Smith AM, Rissel CE. Sex in Australia: Autoerotic, esoteric and other sexual practices engaged in by a representative sample of adults. Australian and New Zealand journal of public health. 2003;27(2):180–90. 10.1111/j.1467-842x.2003.tb00806.x 14696709

[26] Armstrong EA, England P, Fogarty ACK. Accounting for Women’s Orgasm and Sexual Enjoyment in College Hookups and Relationships. American Sociological Review. 2012;77(3):435–62.

[27] Brody S, Costa RM. Satisfaction (Sexual, Life, Relationship, and Mental Health) Is Associated Directly with Penile–Vaginal Intercourse, but Inversely with Other Sexual Behavior Frequencies. The Journal of Sexual Medicine. 2009;6(7):1947–54. 10.1111/j.1743-6109.2009.01303.x 19453891

[28] Tao P, Brody S. Sexual behavior predictors of satisfaction in a Chinese sample. The journal of sexual medicine. 2011;8(2):455–60. 10.1111/j.1743-6109.2010.02129.x 21114768

[29] Fugl‐Meyer KS, Öberg K, Lundberg PO, Lewin B, Fugl‐Meyer A. Epidemiology: On orgasm, sexual techniques, and erotic perceptions in 18‐to 74‐year‐old swedish women. The journal of sexual medicine. 2006;3(1):56–68. 10.1111/j.1743-6109.2005.00170.x 16409218

[30] de Visser RO, Smith AMA, Rissel CE, Richters J, Grulich AE. Sex in Australia: Heterosexual experience and recent heterosexual encounters among a representative sample of adults. Australian and New Zealand Journal of Public Health. 2003;27(2):146–54. 10.1111/j.1467-842x.2003.tb00802.x 14696705

[31] Herbenick D, Reece M, Schick V, Sanders SA, Dodge B, Fortenberry JD. An Event-Level Analysis of the Sexual Characteristics and Composition Among Adults Ages 18 to 59: Results from a National Probability Sample in the United States. The Journal of Sexual Medicine. 2010;7:346–61. 10.1111/j.1743-6109.2010.02020.x 21029390

[32] Herbenick D, Bowling J, Fu T-CJ, Dodge B, Guerra-Reyes L, Sanders S. Sexual diversity in the United States: Results from a nationally representative probability sample of adult women and men. PloS one. 2017;12(7):e0181198. 10.1371/journal.pone.0181198 28727762 PMC5519052

[33] Rehor JE. Sensual, erotic, and sexual behaviors of women from the “kink” community. Archives of sexual behavior. 2015;44(4):825–36. 25795531 10.1007/s10508-015-0524-2 PMC4379392

[34] Richters J, de Visser RO, Badcock PB, Smith AM, Rissel C, Simpson JM, et al. Masturbation, paying for sex, and other sexual activities: the Second Australian Study of Health and Relationships. Sexual health. 2014;11(5):461–71. 10.1071/SH14116 25376999

[35] Swieczkowski JB, Walker CE. Sexual behavior correlates of female orgasm and marital happiness. Journal of Nervous and Mental Disease. 1978. 10.1097/00005053-197805000-00004 650197

[36] Pierce AP. The coital alignment technique (CAT): An overview of studies. Journal of sex & marital therapy. 2000;26(3):257–68. 10.1080/00926230050084650 10929574

[37] Krejčová L, Kuba R, Flegr J, Klapilová K. Kamasutra in Practice: The Use of Sexual Positions in the Czech Population and Their Association With Female Coital Orgasm Potential. Sexual Medicine. 2020;8(4):767–76. 10.1016/j.esxm.2020.07.003 32800750 PMC7691886

[38] Towne A. Clitoral stimulation during penile-vaginal intercourse: A phenomenological study exploring sexual experiences in support of female orgasm. The Canadian Journal of Human Sexuality. 2019;28(1):68–80.

[39] Chatton D, Desjardins J-Y, Desjardins L, Tremblay M. La sexologie clinique basée sur un modèle de santé sexuelle. Psychothérapies. 2005;25(1):3–19.

[40] Salisbury CM, Fisher WA. “Did you come?” A qualitative exploration of gender differences in beliefs, experiences, and concerns regarding female orgasm occurrence during heterosexual sexual interactions. The Journal of Sex Research. 2014;51(6):616–31. 10.1080/00224499.2013.838934 24350619

[41] Cabrera C, Ménard AD. “She exploded into a million pieces”: a qualitative and quantitative analysis of orgasms in contemporary romance novels. Sexuality & Culture. 2013;17(2):193–212.

[42] Braun V, Kitzinger C. “Snatch,”“Hole,” or “Honey‐pot”? Semantic categories and the problem of nonspecificity in female genital slang. Journal of Sex Research. 2001;38(2):146–58.

[43] Gordon M. Sexual slang and gender. Women and Language. 1993;16(2):16–22.

[44] McCormick NB. Sexual scripts: Social and therapeutic implications. Sexual and Relationship Therapy. 2010;25(1):96–120.

[45] Bakaroudis M. Outercourse: Exploring nonpenetrative forms of pleasurable safer sex. American Journal of Sexuality Education. 2014;9(3):381–97.

[46] Marcus JL, Snowden JM. Words Matter: Putting an End to "Unsafe" and "Risky" Sex. Sexually transmitted diseases. 2020;47(1):1–3. 10.1097/OLQ.0000000000001065 31517770 PMC6953392

[47] Whipple B. G spot. The International Encyclopedia of Human Sexuality. 2015:427–500.

[48] Opperman E, Braun V, Clarke V, Rogers C. “It Feels So Good It Almost Hurts”: Young Adults’ Experiences of Orgasm and Sexual Pleasure. Journal of Sex Research. 2014(ahead-of-print):1–13. 10.1080/00224499.2012.753982 23631739

[49] Herbenick D, Fu T-C, Arter J, Sanders SA, Dodge B. Women’s experiences with genital touching, sexual pleasure, and orgasm: Results from a US probability sample of women ages 18 to 94. Journal of sex & marital therapy. 2018;44(2):201–12. 28678639 10.1080/0092623X.2017.1346530

[50] Herbenick D, Bartelt E, Fu T-C, Paul B, Gradus R, Bauer J, et al. Feeling scared during sex: findings from a US probability sample of women and men ages 14 to 60. Journal of Sex & Marital Therapy. 2019;45(5):424–39. 30946623 10.1080/0092623X.2018.1549634

[51] Hensel DJ, Schick V, Herbenick D, Dodge B, Reece M, Sanders SA, et al. Lifetime Lubricant Use among a Nationally Representative Sample of Lesbian‐and Bisexual‐Identified Women in the U nited S tates. The Journal of Sexual Medicine. 2015;12(5):1257–66. 10.1111/jsm.12873 25974238

[52] La France BH. Predicting sexual satisfaction in interpersonal relationships. Southern Communication Journal. 2010;75(3):195–214.

[53] Blunt-Vinti H, Jozkowski KN, Hunt M. Show or tell? Does verbal and/or nonverbal sexual communication matter for sexual satisfaction? Journal of sex & marital therapy. 2019;45(3):206–17. 10.1080/0092623X.2018.1501446 30040593

[54] Hess JA, Coffelt TA. Verbal communication about sex in marriage: Patterns of language use and its connection with relational outcomes. Journal of sex research. 2012;49(6):603–12. 10.1080/00224499.2011.619282 22004117

[55] Frederick DA, Lever J, Gillespie BJ, Garcia JR. What keeps passion alive? Sexual satisfaction is associated with sexual communication, mood setting, sexual variety, oral sex, orgasm, and sex frequency in a national US study. The Journal of Sex Research. 2017;54(2):186–201. 10.1080/00224499.2015.1137854 26900897

[56] Ziherl S, Masten R. Differences in predictors of sexual satisfaction and in sexual satisfaction between female and male university students in Slovenia. Psychiatria Danubina. 2010;22(3):425–9. 20856186

[57] Milstein S, Hilliard TE, Hall S, Knox D, Hunter G. Factors That Impact College Students’ Perceptions of Sexual Pleasure and Satisfaction. American Journal of Sexuality Education. 2020;15(1):99–110.

[58] Trompeter SE, Bettencourt R, Barrett-Connor E. Sexual activity and satisfaction in healthy community-dwelling older women. The American journal of medicine. 2012;125(1):37–43. e1. 10.1016/j.amjmed.2011.07.036 22195529 PMC3246190

[59] Shahhosseini Z, Gardeshi ZH, Pourasghar M, Salehi F. A review of affecting factors on sexual satisfaction in women. Materia socio-medica. 2014;26(6):378. 10.5455/msm.2014.26.378-381 25685081 PMC4314168

[60] Braun V, Clarke V. Using thematic analysis in psychology. Qualitative research in psychology. 2006;3(2):77–101.

[61] Vaismoradi M, Turunen H, Bondas T. Content analysis and thematic analysis: Implications for conducting a qualitative descriptive study. Nursing & health sciences. 2013;15(3):398–405. 10.1111/nhs.12048 23480423

[62] Hensel DJ, Herbenick D, Beckmeyer JJ, Fu T-c, Dodge B. Adolescents’ Discussion of Sexual and Reproductive Health Care Topics With Providers: Findings From a Nationally Representative Probability Sample of US Adolescents. Journal of Adolescent Health. 2020. 10.1177/1363459318785696 32788074

[63] Perkins R. 2018 OMGYES Women’s Pleasure Study-Angling, Rocking, Shallowing and Pairing Items. Ann Arbor, MI: Inter-university Consortium for Political and Social Research [distributor]; 2020.

[64] Gruskin S, Kismödi E. A Call for (Renewed) Commitment to Sexual Health, Sexual Rights, and Sexual Pleasure: A Matter of Health and Well-Being. American journal of public health. 2020;110(2):159. 10.2105/AJPH.2019.305497 31913674 PMC6951372

[65] Cacchioni T. Heterosexuality and’the labour of love’: A contribution to recent debates on female sexual dysfunction. Sexualities. 2007;10(3):299–320.

[66] Frith H. Labouring on orgasms: embodiment, efficiency, entitlement and obligations in heterosex. Culture, health & sexuality. 2013;15(4):494–510. 10.1080/13691058.2013.767940 23464744

