# Supplementary material for: Women’s techniques for making vaginal penetration more pleasurable: Results from a nationally representative study of adult women in the United States
Source: PLoS One. 2021 Apr 14;16(4):e0249242. doi: 10.1371/journal.pone.0249242 (PMC8046227; doi:10.1371/journal.pone.0249242)
Supplement: S1 Table — (DOCX) [file pone.0249242.s001.docx]

**S1 Table. Definitions for and line drawing illustrations of techniques for enhancing sexual pleasure during vaginal stimulation and penetration**

| **Techniques** | **Definition** | **Example Illustrations** |
| --- | --- | --- |
| **Angling** | Rotating, raising, or lowering the pelvis/hips during penetration to adjust where inside the vagina the toy or penis rubs and what it feels like. | 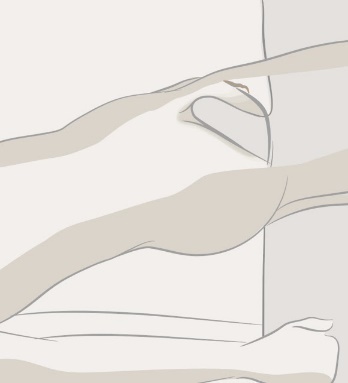 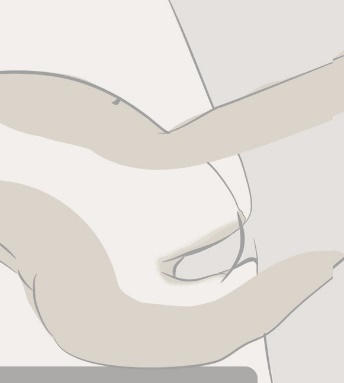 |
| **Rocking** | The base of a penis or sex toy rubbing against the clitoris constantly during penetration, by staying all the way inside the vagina rather than thrusting in and out. | 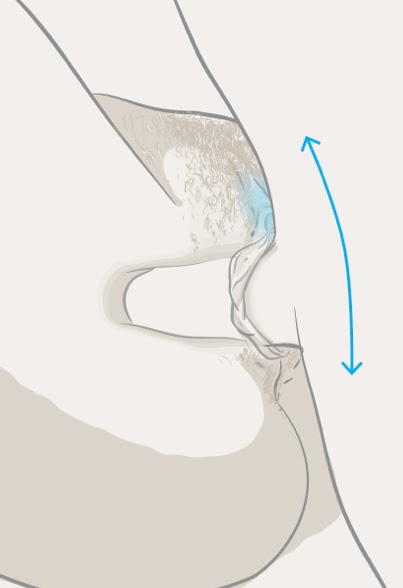 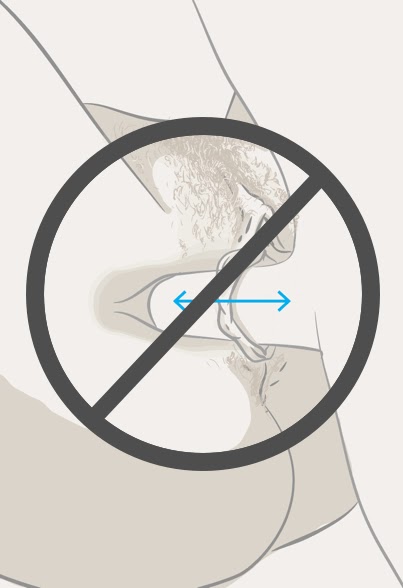 |
| **Shallowing** | Penetrative touch just inside of the entrance of the vagina - not on the outside, but also not deep inside - with a fingertip, sex toy, penis tip, tongue, or lips. | 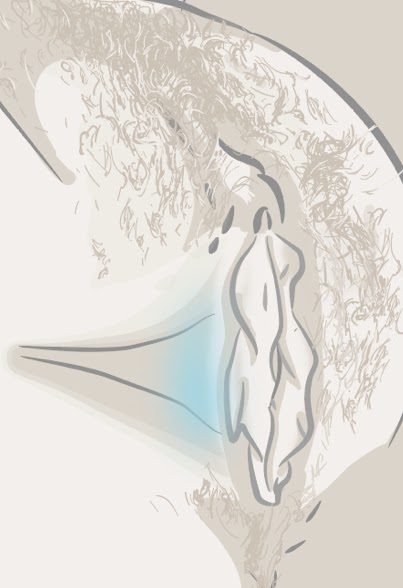 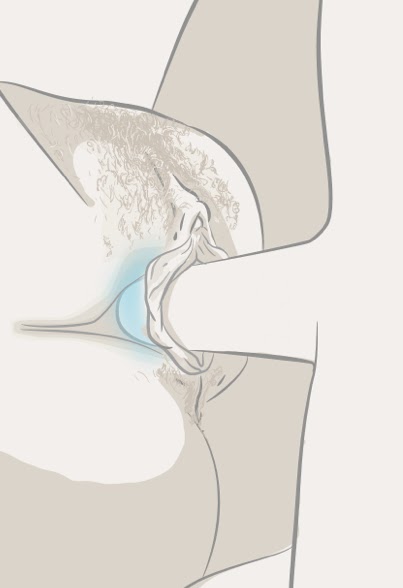 |
| **Pairing** | When a woman herself (Solo Pairing) or her partner (Partner Pairing) reaches down to stimulate her clitoris with a finger or sex toy at the same time as her vagina is being penetrated. | 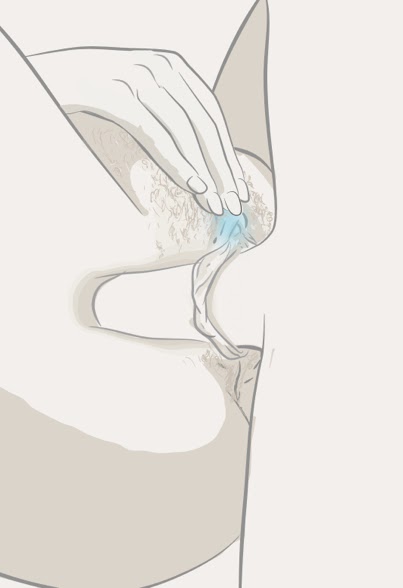 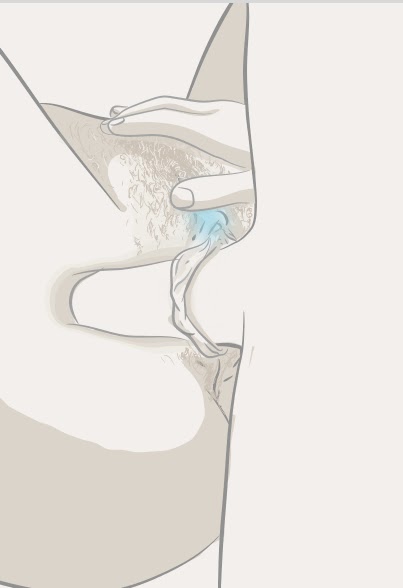  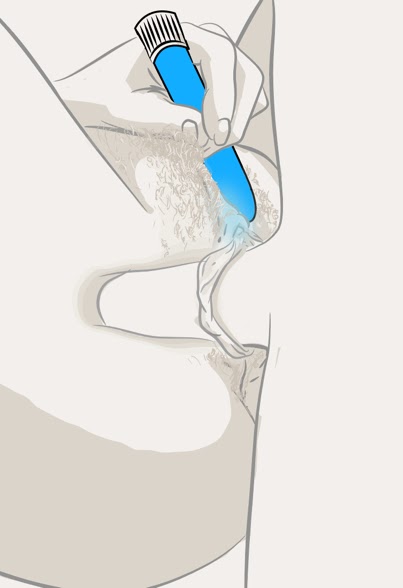 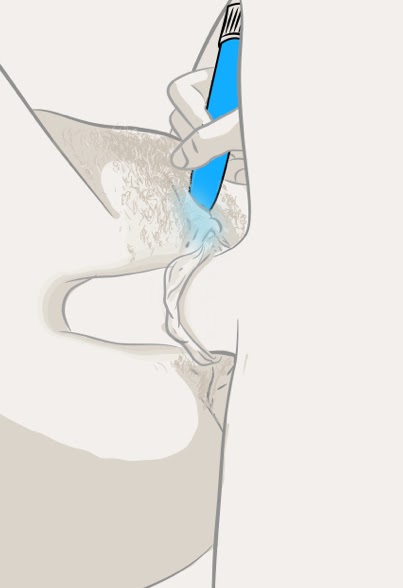 |
