# Supplementary material for: Women’s techniques for making vaginal penetration more pleasurable: Results from a nationally representative study of adult women in the United States
Source: PLoS One. 2021 Apr 14;16(4):e0249242. doi: 10.1371/journal.pone.0249242 (PMC8046227; doi:10.1371/journal.pone.0249242)
Supplement: S1 Appendix — (DOCX) [file pone.0249242.s002.docx]

Selected items from the Second OMGYES Pleasure Report (**note:** demographic items are not included because they were assessed through Ipsos Knowledge Panel profile data)

**Angling items:**

“Many women have discovered that, during vaginal intercourse with a toy or penis, the angle or position of their hips makes a big difference in where the toy or penis rubs and what it feels like.

Thinking back over your entire life, which of the following ways of adjusting or angling your hips have you used to make sex better / more pleasurable for you during penetration?”

1. Rotating your hips downward while on your back
   1. Yes
   2. No
2. Rotating your hips upward while lying on your stomach
3. Yes
4. No
5. Rotating your hips upward while lying on your back
6. Yes
7. No
8. Rotating your hips downward while lying on your stomach
9. Yes
10. No
11. Putting a pillow under your butt so your pelvis is higher
12. Yes
13. No
14. Putting a pillow under your lower back so your pelvis is lower
15. Yes
16. No

**Rocking items:**

Thinking back over your entire life, how pleasurable have you found the following ways of having vaginal penetration?

| **Statement** | **1. Not at all pleasurable** | **2. A little pleasurable** | **3. Somewhat pleasurable** | **4. Very pleasurable** | **5. Don’t know or never tried** |
| --- | --- | --- | --- | --- | --- |
| **1.1** A partner’s penis ‘staying inside’ with their lower body staying in contact and pressing against your clitoris |  |  |  |  |  |
| **1.2** A toy (vibrator or other object) ‘staying inside’ your vagina without in-and-out thrusting |  |  |  |  |  |

**Shallowing items:**

“These questions are about how it feels to be touched just at the entrance to the vagina.

**1.** Thinking back over your entire life, how pleasurable have you found the following kinds of touch just inside, at the entrance of your vagina (just shallow penetration - not on the outside, but also not deep inside)?”

| **Statement** | **1. Not at all pleasurable** | **2. A little pleasurable** | **3. Somewhat pleasurable** | **4. Very pleasurable** | **5. Don’t know or never tried** |
| --- | --- | --- | --- | --- | --- |
| **1.1** A fingertip touching just barely inside the entrance to your vagina |  |  |  |  |  |
| **1.2** A sex toy touching just barely inside the entrance to your vagina |  |  |  |  |  |
| **1.3** A penis tip touching just barely inside the entrance to your vagina |  |  |  |  |  |
| **1.4** A mouth/tongue touching just barely inside the entrance to your vagina |  |  |  |  |  |

**[Skip logic: If 1.1 or 1.2 or 1.3 or 1.4 rated 2-4, then ask Q2 - otherwise skip to next section]**

**2.** “Some women say that having the tip of a penis, sex toy, or fingers just inside the entrance to the vagina (with shallow penetration), even briefly, changes how pleasurable the sex or sexual touching that follows feels.”

1. The penetration that comes next is more likely to be pleasurable
2. Yes
3. No
4. The orgasm that comes next is more likely to be stronger or more intense
5. Yes
6. No
7. The orgasm that comes next is more likely to happen at all
8. Yes
9. No
10. It depends -- there is no consistent pattern
11. Yes
12. No

**Pairing items:**

Which, if any, of the following has helped you to orgasm more often or have more pleasurable sex than penetration on its own?

1. Penetration while stimulating my own clitoris at the same time with a finger
2. Yes
3. No
4. Penetration while stimulating my own clitoris at the same time with a toy or vibrator
5. Yes
6. No
7. Penetration with my partner stimulating my clitoris at the same time with a finger
8. Yes
9. No
10. Penetration with my partner stimulating my clitoris at the same time with a toy or vibrator
11. Yes
12. No
